# Detection of Antibodies Against the SARS-CoV-2 Spike Protein and Analysis of the Peripheral Blood Mononuclear Cell Transcriptomic Profile, 15 Years After Recovery From SARS

**DOI:** 10.3389/fcimb.2021.768993

**Published:** 2021-11-18

**Authors:** Lili Zhao, Na Han, Yali Zheng, Huiying Rao, Jia Li, Yanwen Chen, Bing Yu, Yu Xu, Hongsong Chen, Zhancheng Gao, Baoguo Jiang

**Affiliations:** ^1^ Department of Respiratory and Critical Care Medicine, Peking University People’s Hospital, Beijing, China; ^2^ Department of Central Laboratory, Peking University People’s Hospital, Beijing, China; ^3^ Department of Respiratory and Critical Care Medicine, Xiang’An Hospital of Xiamen University, Xiamen, China; ^4^ Peking University Hepatology Institute and Beijing Key Laboratory of Hepatitis C and Immunotherapy for Liver Diseases, Peking University People’s Hospital, Beijing, China; ^5^ Department of Emergency Medicine, Peking University People’s Hospital, Beijing, China; ^6^ Department of Orthopedics and Trauma, Peking University People’s Hospital, Beijing, China

**Keywords:** SARS-CoV, SARS-CoV-2, COVID-19, transcriptomic profile, immune cross-reactivity

## Abstract

Severe acute respiratory syndrome coronavirus 2 (SARS-CoV-2) shows a high degree of homology with SARS-CoV. They share genes, protein sequences, clinical manifestations, and cellular entry patterns. Thus, SARS research may serve helpful in gaining a better understanding of the current coronavirus disease 2019 (COVID-19) pandemic. Serum antibodies from convalescent patients with SARS collected in 2018 were used to target the recombinant SARS-CoV-2 spike protein *via* a chemiluminescence microsphere immunoassay. Antibodies of convalescent patients with SARS exhibited serous immune cross-reactivity with the SARS-CoV-2 spike protein. The serous antibodies, excluding S22 of convalescent patients with SARS, did not competitively inhibit the binding of SARS-CoV-2 spike protein to ACE2. T cellular immunity research was conducted *in vitro* using peripheral blood mononuclear cells (PBMCs) stimulated by pooled peptide epitopes 15 years post-infection. Interferon gamma was detected and the PBMC transcriptomic profile was obtained. The heatmap of the transcriptomic profile showed that mRNAs and circRNAs of the SARS group clustered together after being stimulated by the peptide epitope pool. Differentially expressed mRNAs were most significantly enriched in immunity and signal transduction (P < 0.01). SARS elicits cytokine and chemokine responses, partially consistent with previously published data about COVID-19. Overall, our results indicate that antibodies from convalescent patients with SARS persisted for 15 years and displayed immune cross-reactivity with the SARS-CoV-2 spike protein. The immune status of patients with SARS 15 years post-infection may provide a better understanding of the future immune status of patients with COVID-19.

## Introduction

Coronavirus disease 2019 (COVID-19) is caused by severe acute respiratory syndrome coronavirus 2 (SARS-CoV-2). The disease is associated with causing 240 million infections and 5 million deaths worldwide since its emergence in December 2019 (World Health Organization). SARS-CoV-2 is highly homologous to SARS-CoV—the causative agent of the severe acute respiratory syndrome (SARS) outbreak of 2003— and shares 79%–82% of its genome and 95%–100% of its proteome with the latter (Lu et al., 2020; Xu et al., 2020).

The SARS outbreak caused by SARS-CoV in 2003 resulted in 8422 probable cases including approximately 919 related deaths (Yang et al., 2020a). The most frequent initial symptoms were fever (100%), cough (61%), myalgia (48%), dyspnoea (40%), diarrhoea (31%), and rigor (30%). Initial laboratory data indicated lymphopenia, thrombocytopenia, and elevated aspartate transaminase, alanine aminotransferase, lactic dehydrogenase, and C-reactive protein levels. Chest radiograph abnormalities suggesting pneumonia were observed in 73% of the patients. During hospitalisation, 90.8% of the patients had respiratory distress and needed oxygen supplementation (Wang J. T. et al., 2004). Furthermore, 40%–45% of the patients with SARS-CoV-2 infection are asymptomatic. However, the clinical manifestations of the symptomatic patients are partially similar to or are worse than those of patients with SARS, with the infected individuals exhibiting a strong capacity for viral spread (Chang et al., 2020; Chen et al., 2020; Huang et al., 2020; Oran and Topol, 2020; Wang et al., 2020). COVID-19 causes a few immune disorders such as interstitial lung injury, coagulopathy, and vasculitis (Agricola et al., 2020; Dominguez-Santas et al., 2020; Oda et al., 2020; Ramlall et al., 2020).

Respiratory epithelial cells are infected by both SARS-CoV and SARS-CoV-2 *via* the receptor binding domain (RBD) of surface spike glycoprotein (S protein). These viruses selectively bind to angiotensin-converting enzyme 2 (ACE2) that acts as a host receptor for viral infection (Aguiar et al., 2020; Ou et al., 2020; Zhu et al., 2020). Thereafter, a series of immune reactions such as a cytokine storm, lymphopenia, and T cell exhaustion are triggered and cascaded.

As SARS-CoV-2 is a newly emerging pathogen, it is currently difficult to elucidate what the immune status of patients would be in the future. Considering the homology between the gene and protein sequences of SARS-CoV and SARS-CoV-2, as well as several similar clinical manifestations and cellular entry patterns, we investigated whether SARS-CoV-2 undergoes an immune cross-reactivity with the sera of convalescent patients with SARS-CoV infection. The convalescent patients were healthcare workers who had recovered from SARS in 2003. In our previous work, a 15-year prospective cohort study to monitor the effects on bones and lungs post-recovery from SARS infection, we had observed femoral head necrosis, abnormal pulmonary function, and interstitial changes as some long-term afflictions. (Zhang et al., 2020). In this study, we explored the immune response of their PBMCs following stimulation by peptide epitopes to mimic a SARS reinfection occurring 15 years later. These results provide hints to predict the immune profile of COVID-19 that may manifest after many years. Our study may contribute to a better understanding of the immune barrier to either SARS-CoV-2 or novel coronavirus infection and to the prevention of further epidemics.

## Material and Methods

### Serum and PBMC Isolation From Patients

Whole blood was collected from convalescent patients with SARS and uninfected close contacts (Control group) between February and May 2018. Written consent was obtained from all enrolled personnel before sample collection. All collected whole blood samples were placed in 1 vacuum coagulation blood vessel and 2 heparin-coated blood vessels, and then centrifuged for 10 min at 2000 rpm to separate the cellular fraction, serum, and plasma. All samples were aliquoted and stored at −80°C. PBMCs were isolated *via* density-gradient sedimentation using Ficoll–Paque (Sigma, USA) according to a previously described method (Weiskopf et al., 2013). The isolated PBMCs were cryopreserved in RPMI 1640 cell culture media containing 10% foetal bovine serum (FBS) (Gibco, USA) and 10% dimethyl sulfoxide (DMSO; Sigma, USA), and stored in liquid nitrogen until required for the assays.

### Microsphere-Based Antibody Assay

Serum antibodies from convalescent patients with SARS, collected in 2018, were detected using a commercially available kit (Innodx, Fujian, China) *via* a chemiluminescence microsphere immunoassay. A mixture of 50 µL of serum sample, 50 µL of SARS-CoV-2 recombinant spike protein-coated magnetic particles, and 50 µL of reaction diluent was incubated in a reaction cup at 37°C for 15 min. After washing, removing unbound antibodies, and washing again, 100 µL of pre-excitation solution and 100 µL of excitation solution were added to the mixture. An automatic chemiluminescence instrument (Wan200+; UMIC, Fujian, China) was used to detect the relative luminescence unit and calculate the signal-to-cutoff (S/CO). A S/CO > 1 indicates the presence of SARS-CoV-2 antibodies in the body, whereas S/CO ≤ 1 means that the body is devoid of SARS-CoV-2 antibodies.

### ACE2 Competition Assay

ACE2 competition assay was performed using the SARS-CoV-2 ACE2 Competition Assay ELISA kit (KangLang, Shanghai, China). This assay employs the competitive inhibition enzyme immunoassay technique. The microtiter plate provided in this kit had been pre-coated with RBD of the SARS-CoV-2 spike protein. The standard of neutralising antibody was prepared as a 2-fold dilution series with 10000 ng/mL as the highest standard and S0 served as the zero standard (0ng/mL). Serum of convalescent patients with SARS was diluted 20-fold. It was added to the appropriate microtiter plate wells with horseradish peroxidase (HRP)-conjugated ACE2. The competitive inhibition reaction was initiated between HRP-ACE2 and SARS-CoV-2 neutralising antibody in samples. TMB substrate solution was added to the wells; the colour developed inversely proportional to the amount of SARS-CoV-2 neutralising antibody in the sample. The colour development was stopped and the intensity of the colour was measured at 450nm; the correction wavelength was set at 570nm. The cut-off was 1/2 ODS0. OD of samples <1/2 ODS0 was considered to have an ACE2 competition and that of sample ≥1/2 ODS0 was considered no ACE2 competition.

### Synthetic Peptide Pool

SARS-CoV virus-specific CD4 and CD8 peptide epitopes were synthesised by Genscript (Jiangsu, China). Sequence information for the selected peptides was derived from peptides reported to be effective, as per previous studies (He et al., 2004; Wang Y. D. et al., 2004; Zhao et al., 2007; Janice Oh et al., 2012). All peptides were purified *via* high-pressure liquid chromatography with purities higher than 80%. The lyophilised peptides were stored in aliquots at −80°C.

### *In Vitro* Stimulation of 66 Peptides

The 66 peptides were dissolved in DMSO at 5 mg/mL, aliquoted, and stored at −20°C. PBMCs from convalescent patients with SARS and control donors were cultured in a six-well plate with RPMI 1640 medium containing 10% FBS and 100 U/mL recombinant human interleukin (IL)-2 (rhIL-2). The next day, the 66 peptide-DMSO pool (50 µg/mL) was added to the medium, which was termed the 66p group. In contrast, only DMSO was added to the background control, which was termed the DMSO group. Half of the medium was changed on days 3 and 5, with supplementation of the 66 peptides (50 µg/mL) and rhIL-2 (100 U/mL). On day 7, the cells were harvested to test either for the presence of peptide-specific CD8 T cells *via* an interferon-release ELISPOT assay or for total RNA extraction.

### ELISPOT Assay

An ELISPOT assay was performed using a commercially available kit (Dakewe, China). A cell suspension was added to an interferon gamma (IFN-γ) monoclonal antibody pre-coated 96-well plate at 5×10^4^ cells per well. Next, 1× PHL-A was added to the positive control well, whereas rhIL-2 (100 U/mL) and the 66 peptides (50 µg/mL) were added to the experimental well, and rhIL-2(100 U/mL) and DMSO were added to the negative control well. The 96-well plate was incubated at 37°C under 5% CO_2_ for 20 h. The cells were lysed with hypotonic deionised water for 10 min; then, 100 µL/well biotin-labelled antibodies was added after washing, and the preparation was incubated at 37°C for 1 h. The plate was washed and 100 µL/well AEC colour developing solution was added and left to stand for 20 min in the dark. Next, the preparation was washed with deionised water to stop colour development. Finally, brown coloured spots, which represented epitope-specific IFN-γ-derived T cells, were counted using an automatic ELISPOT reader.

### Total RNA Extraction and GeneChip Arrays

The total RNA was extracted from stimulated PBMCs using TRIzol reagent (Life Technology, USA) and a RNeasy Mini Kit (Qiagen, USA). Biotinylated cDNAs were prepared according to the standard Affymetrix protocol with 150 ng of total RNA using an Ambion^®^ WT Expression Kit. Next, the labelled cDNA was hybridised for 16 h at 45°C on GeneChip Affymetrix Human Clariom^®^ D Array (Affymetrix). GeneChips were washed and stained in Affymetrix Fluidics Station 450. All arrays were scanned using the Affymetrix^®^ GeneChip Command Console in the GeneChip^®^ Scanner 3000 7G. The data were analysed using a robust multichip analysis (RMA) algorithm that utilises Affymetrix global scaling and default analysis settings as a normalisation method. Values are presented as log2 RMA signal intensity. Differentially expressed genes between the groups were assessed using Student’s *t*-test. The threshold set for upregulated and downregulated genes were a fold change (FC) > 2.0 and a P value < 0.05.

### Reverse Transcription and Real-Time PCR of mRNA

Real-time PCR was used to verify the differential expression of mRNA. Thereafter, cDNA was synthesised using the PrimeScript™ RT reagent Kit (Takara, Japan) with 200 ng of total RNA from the same samples as those used in the microarray. Real-time PCR was performed using TB Green^®^ Premix Ex Taq™ (Takara, Japan) according to the manufacturer’s instructions. The primers used are listed in **Supplementary Table 4**. PCR amplification of each sample was performed in duplicates. Gene expression levels were quantified relative to the expression of GAPDH using the 2^-△△Ct^ method. The differences in gene expression levels between the groups were compared using Student’s *t*-test. Statistical significance was set at P < 0.05.

### Cluster Analysis

Hierarchical clustering was performed based on differentially expressed mRNAs and circRNAs using web-based online bioinformatics resource (https://www.metaboanalyst.ca/).

### Functional Enrichment Analysis of mRNA

DAVID (https://david.ncifcrf.gov/), a freely accessed web-based online bioinformatics resource, was used to perform Kyoto Encyclopedia of Genes and Genomes (KEGG) pathway enrichment analysis and Gene Ontology (GO) term enrichment analysis pertaining to differently expressed mRNAs with a setting of P < 0.05 and counts > 2.0

### MicroRNA Target Prediction

Target microRNAs of mRNAs and circRNAs were predicted using Miranda. Miranda predicts biological targets of mRNAs and circRNAs *via* a dynamic programming sequence alignment SW algorithm and free energy calculation. When the score value and free energy of sequence alignment are both higher than the threshold, the miRNAs are considered to target circRNAs as compared to miRNAs (Myers and Miller, 1989).

### Construction of a circRNA–miRNA–mRNA Network

A circRNA–miRNA–mRNA regulatory network was constructed using Cytoscape software (version: 3.8.2).

### Statistical Analysis

Statistical analyses were conducted using GraphPad Prism 8.0. Statistical details of the experiments are provided in the respective figure legends. The histogram is expressed in terms of mean and standard error (SE). Normally distributed continuous variables were expressed as means ± standard error from the mean, and non-normally distributed continuous variables were expressed as medians and interquartile ranges. Two groups of equivalent continuous variables with normal distributions were compared using Student’s *t*-test. Non-normal continuous variables or variables with unequal variances between groups were compared using Mann–Whitney U test. Results with a two-sided P value < 0.05 were considered statistically significant. *P < 0.05, **P < 0.01, ***P < 0.001, ****P < 0.0001.

## Results

### Characteristics of the SARS and Control Group in 2018

The clinical characteristics of the enrolled convalescent patients with SARS (n=53) and control population (n=45) are presented in **Table 1**. All data were collected in 2018. There were no significant differences between the groups in terms of sex, age, comorbidities, and laboratory testing except pulmonary fibrosis. The number of pulmonary fibrosis cases was significantly higher in the SARS convalescent group than the control group (P<0.0001). Pulmonary fibrosis was caused by SARS in 2003.

**Table 1:** Clinical characteristics and laboratory findings of the SARS and Control groups in 2018.

	SARS n = 53	Control n = 45	P value
**Male sex (%)**	8 (15.09%)	10 (22.22%)	0.364
**Age (years)**	46 (40-51)	44 (40-48)	0.337
**Comorbidities, n (%)**			
**Heart disfunction**	1 (2.0%)	1 (2.4%)	1.000
**Chronic renal disease**	0 (0.0%)	0 (0.0%)	–
**Liver disease**	0 (0.0%)	0 (0.0%)	–
**Diabetes mellitus**	2 (4.1%)	2 (4.8%)	1.000
**High pressure**	6 (12.2%)	5 (11.9%)	0.960
**Pulmonary fibrosis**	18 (36.7%)	0 (0.0%)	**<0.0001**
**Laboratory findings**			
**White blood cell (×10^3^/mm^3^)**	5.72 ± 0.24	5.89 ± 0.21	0.614
**Neutrophils(×10^3^/mm^3^)**	3.19 ± 0.16	3.26 ± 0.15	0.740
**Lymphocytes(×10^3^/mm^3^)**	1.74 (1.41-2.34)	1.93 (1.68-2.28)	0.420
**Hemoglobin level (g/dL)**	135.36 ± 2.00	138.73 ± 2.30	0.269
**Platelet count (×10^3^/mm^3^)**	269.00 (230.50-309.50)	252.00 (234.00-293.50)	0.408
**Glucose (mmol/L)**	5.09 (4.78-5.41)	5.21 (4.81-5.64)	0.0.32
**Albumin (g/L)**	44.28 ± 0.32	45.36 ± 0.36	0.073
**ALT (U/L)**	17.00 (12.00-21.50)	17.00 (13.00-23.00)	0.892
**AST (U/L)**	18.00 (16.00-24.00)	18.00 (16.00-21.00)	0.429
**LDH (U/L)**	159.13 ± 3.34	165.84 ± 4.00	0.197
**CREA (µmol/L)**	56.00 (52.00-56.50)	54.00 (47.00-64.00)	0.175
**IgA (g/L)**	2.05 (1.69-3.05)	2.17 (1.56-2.54)	0.503
**IgG (g/L)**	12.30(10.90-13.45)	13.00 (11.20-14.65)	0.059
**IgM (g/L)**	0.95 (0.71-1.15)	1.09 (0.76-1.37)	0.163
**C3 (g/L)**	0.92 ± 0.02	0.95 ± 0.03	0.417

SARS group: convalescents who were infected with SARS-CoV in 2003; Control group: close contacts of SARS group who were not infected with SARS-CoV in 2003. Data are presented as means ± standard error from the mean, or median (interquartile range) or n (%). ALT, Alanine aminotransferase; AST, Aspartate aminotransferase; LDH, lactate dehydrogenase; CREA, Creatinine; IgA, immunoglobin A; IgG, immunoglobin G; IgM, immunoglobin M; C3, complement C3. The bold value mean that P value < 0.05 and were statistically significant.

### Immune Cross-Reactivity Between Serum Collected From Convalescent Patients With SARS 15 Years Post-Infection and SARS-CoV-2 Infection

**Figure 1:**
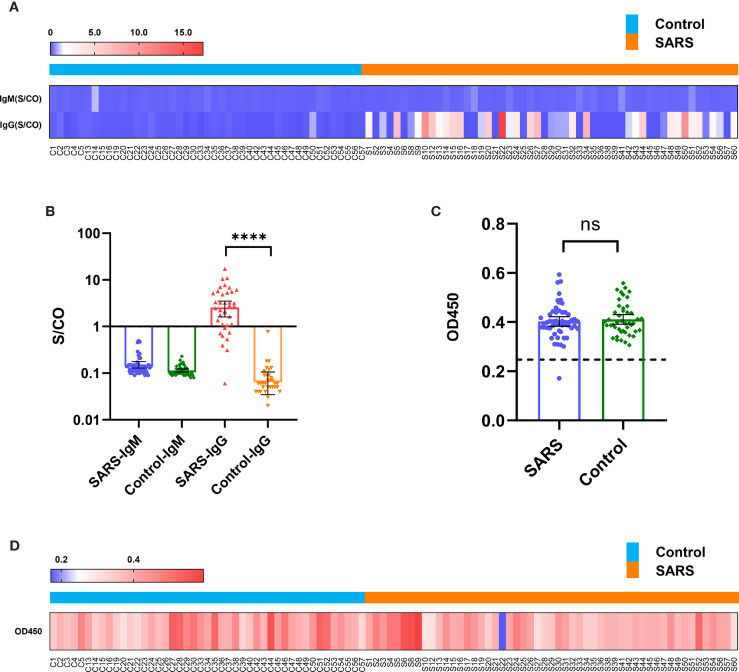
Immune cross-reactivity of convalescent patient serums with SARS collected from 15 years post-infection against the SARS-CoV-2 spike protein. **(A)** Heatmap of antibody titres. **(B)** Histogram of antibody titres expressed in terms of mean and 95% confidence interval (CI). **(C)** Histogram of OD450 in the ACE2 competition assay in terms of mean and 95% CI. **(D)** Heatmap of OD450 in the ACE2 competition assay. ****P < 0.0001. ns, no significant difference.

Fifty-three convalescent patients infected with SARS-CoV in 2003 and 45 close contacts who were not infected with SARS-CoV in 2003, but sampled for sera 15 years later in 2018 (control), were tested. In 2020, antibodies targeting the spike protein of SARS-CoV-2 in both groups were detected using the chemiluminescence microsphere immunoassay. The results showed that the control population was negative for SARS-CoV-2 immunoglobulin (Ig) M and IgG. Among the serum samples collected from convalescent patients with SARS, 29 (55%) samples were positive for SARS-CoV-2 IgG antibodies, whereas all were negative for IgM (**Figures 1A, B** and **Supplementary Table 1**). This suggested that antibodies targeting SARS-CoV persisted 15 years and displayed immune cross-reactivity between SARS-CoV and SARS-CoV-2.

### ACE2 Competition Assay

The serous ACE2 competition assay showed that the samples were negative excluding S22 of convalescent patients with SARS (**Figures 1C, D** and **Supplementary Table 2**). S22 exhibited the highest antibody titre in the microsphere-based antibody assay (**Figure 1A**). This result suggested that only a few people could produce protective antibodies, which prevent SARS-CoV-2 reinfection by competitively inhibiting the binding of SARS-CoV-2 spike protein to ACE2.

### Characteristics of the 53 Patients With SARS in 2003

Convalescents patients with SARS were divided into two groups according to the S/CO value of SARS-CoV-2 IgG *via* a chemiluminescence microsphere immunoassay (**Figure 1A**); S/CO > 1 represents the presence of IgG antibodies and S/CO < 1 represents the absence of IgG antibodies. The characteristics and treatments of SARS in 2003 were retrospectively analysed. There were no significant differences in the other indicators between the groups, except rigor (P=0.039) and thymosin therapy (P=0.038) (**Table 2**).

**Table 2:** Clinical characteristics and treatments of SARS in 2003.

	S/CO>1n = 29	S/CO ≤ 1n = 24	P value
**Male sex (%)**	3 (10.3%)	5 (20.8%)	0499
**Age (years)**	47.12 ± 1.81	47.3 9± 1.69	0.914
**Clinical symptoms, n (%)**			
** Fever**	29 (100%)	24 (100%)	–
** Duration of fever(days)**	9.00 (6.50-14.50)	7.00 (5.00-15.00)	0.798
** Rigor**	21 (77.8%)	12 (50%)	**0.039**
** Myalgia**	21 (77.8%)	13 (54.2%)	0.074
** Cough**	14 (51.9%)	12 (50%)	0.895
** Dyspnoea**	17 (63%)	14 (58.3%)	0.735
** Vomiting**	6 (22%)	2 (8.3%)	0.329
** Diarrhoea**	5 (18.5%)	4 (16.7%)	1.000
**Treatments**			
** Total Methylprednisolone (mg)**	3500 (2240-9872)	4575 (2350-15000)	0.491
** Immunoglobulin**	12 (44.4%)	6 (25%)	0.228
** Thymosin**	21 (77.8%)	12 (50%)	**0.038**
**Severity**			
** ARDS**	9 (33.3%)	4 (16.7%)	0.173
** Lung injury**	11 (37.9%)	7 (29.2%)	0.502
** Bone injury**	5 (17.2%)	8 (33.3%)	0.175
** SCAP**	12 (41.4%)	10 (41.7%)	0.983

S/CO>1: presence of SARS-CoV-2 IgG antibodies via a chemiluminescence microsphere immunoassay; S/CO ≤ 1: devoid of SARS-CoV-2 antibodies via a chemiluminescence microsphere immunoassay; Data are presented as means ± standard error from the mean, or median (interquartile range) or n (%). ARDS, acute respiratory distress syndrome; SCAP, severe community acquired pneumonia. The bold value mean that P value <0.05 and were statistically significant.

Univariate and multivariate logistic regression models was used to investigate associations between characteristics and IgG positivity (**Supplementary Table 3**). In the univariate analysis, a significant predictive ability was found for IgG positivity with rigor [OR (95% CI): 0.285 (0.086–0.958)] and thymosin [OR (95% CI): 0.286 (0.085–0.958)], other characteristics showed no significant association. However, the significance disappeared after adjusting for age, sex, fever, myalgia, cough, dyspnoea, vomiting, diarrhoea, methylprednisolone, immunoglobulin, acute respiratory distress syndrome (ARDS), lung injury, bone injury, and severe community acquired pneumonia (SCAP) in multivariate logistic regression model.

### Pool of the 66 Peptide and T Cell Immune Response

**Figure 2:**
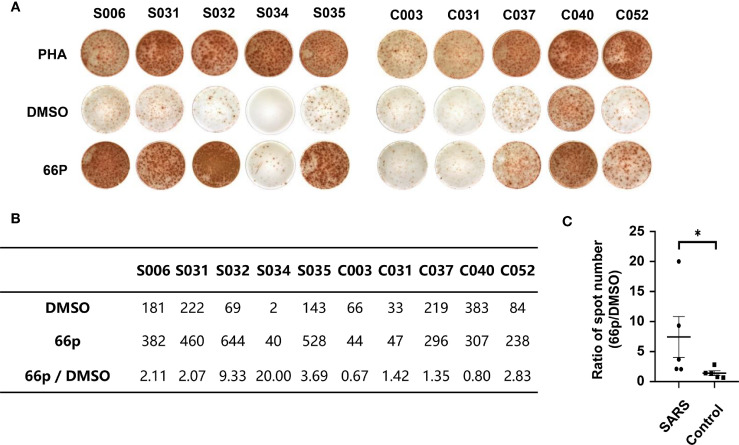
T-cell immune response detected using the IFN-γ ELISPOT assay. **(A)** ELISPOT assay of PBMCs obtained from convalescent patients with SARS and control populations. PHA and DMSO were the positive and negative controls, respectively. **(B)** Table containing spot count from **(A)**. **(C)** Graph depicting statistical analysis of **(B)**; *P < 0.05.

To evaluate immune responses of convalescent patients with SARS 15 years post-infection, a pool of 66 short peptides that contained 9–18 amino acids and covered SARS-CoV replicase, spike, Orf3, Orf4, Orf7, Orf13, envelope, membrane, and nucleocapsid genes was synthesised (**Table 3**). These T cell peptide epitopes are capable of stimulating an immune response in PBMCs. We randomly thawed PBMCs isolated from 5 convalescent patients with SARS (S006, S031, S032, S034, and S035) and 5 controls (C003, C030, C037, C040, and C052) from 2018. After stimulating the PBMCs with the 66 peptide pool, an IFN-γ ELISPOT assay was used to detect T cell immune response. The ELISPOT assay was performed on the following groups: phytohaemagglutinin (PHA) with a universal T cell stimulator as the positive control; the DMSO group (DMSO was the solvent used in the 66 peptide pool) as the negative control; and the 66 peptide pool dissolved in DMSO, termed 66p, as the experimental group. The spot ratio of 66P-DMSO/DMSO in the SARS group was higher than that in the control group (P < 0.05; **Figure 2**). This suggested that the 66 peptide pool stimulated an effective T cell immune response. When the synthesised peptides were aligned with the SARS-CoV-2 proteins, the results showed that more than half of the peptides (34 out of 66) had identical sequences that were found in both SARS-CoV and SARS-CoV-2 (**Table 3**). This suggests that there may exist a T cell cross-reactivity that can help protect the host against SARS-CoV-2 infection.

**Table 3:** Synthesised SARS-CoV peptide epitopes and aligned to proteins of SARS-CoV-2.

Number	Gene	Sequence	Residue	Aligned to proteins of SARS-CoV-2
1	Replicase	TCGYLPTNAVVKMPC	356-370	no
2	Replicase	LLATNNVFRLKGGAP	806-820	no
3	Replicase	VTEFACVVAEAVVKT	869-883	no
4	Replicase	EPVNQFTGYLKLTDN	1001-1015	no
5	Replicase	ARFPKSDGTGTIYTE	4170-4186	SARS-CoV-2 (4199-4213)
6	Replicase	QRLTKYTMADLVYAL	4486-4500	SARS-CoV-2 (4514-4528)
7	Replicase	GNCDTLKEILVTYNC	4506-4520	SARS-CoV-2 (4534-4548)
8	Replicase	KIFVDGVPFVVSTGY	4701-4715	SARS-CoV-2 (4732-4746)
9	Spike	GVYYPDEIFRSDTLYL	39-54	no
10	Spike	FKNKDGFLYVYKGYQPI	187-203	no
11	Spike	IYQTSNFRVVPSGDVVRF	299-316	no
12	Spike	KKISNCVADYSVLYNSTF	343-360	SARS-CoV-2 (38-55)
13	Spike	CYGVSATKL	366-374	SARS-CoV-2 (379-387)
14	Spike	KLPDDFMGCVCVLAWNTR	411-426	no
15	Spike	NIDATSTGNYNYKYRYLR	427-444	no
16	Spike	NYNYKYRYLRHGKLRPF	435-451	no
17	Spike	GPKLSTDLIKNQCVNFNF	513-530	no
18	Spike	IKNQCVNFNFNGLTGTGV	521-538	no
19	Spike	NFNGLTGTGVLTPSSKRF	528-545	no
20	Spike	GVLTPSSKRFQPFQQFGR	536-553	no
21	Spike	WRIYSTGNNVFQTQAGCL	619-636	no
22	Spike	AGCLIGAEHVDTSYECD	633-649	no
23	Spike	DIPIGAGICASYHTVSLL	649-666	no
24	Spike	LLRSTSQKSIVAYTMSL	665-681	no
25	Spike	KSIVAYTMSLGADSSIAY	672-689	no
26	Spike	VKQMYKTPTLKYFGGFNF	767-784	no
27	Spike	ILPDPLKPT	787-795	no
28	Spike	PLKPTKRSFIEDLLFNKV	791-808	no
29	Spike	VLPPLLTDDMIAAYTAAL	842-859	no
30	Spike	VLYENQKQIANQFNKAI	897-913	no
31	Spike	ESLTTTSTALGKLQDVV	918-934	no
32	Spike	EAEVQIDRLITGRLQSL	970-986	SARS-CoV-2 (988-1004)
33	Spike	VVFLHVTYV	1042-1050	SARS-CoV-2 (1060-1068)
34	Spike	PAICHEGKAYF	1061-1071	no
35	Spike	TSWFITQRNFFSPQII	1082-1097	no
36	Spike	RLNEVAKNL	1167-1175	SARS-CoV-2 (1182-1190)
37	Spike	IAGLIAIV	1203-1210	SARS-CoV-2 (1221-1228)
38	Orf3	RFFTLGSITAQPVKI	6-20	no
39	Orf3	PLQASLPFGWLVIGV	36-50	SARS-CoV-2 (36-50)
40	Orf3	CRIIMRCWLCWKCKS	121-135	SARS-CoV-2 (122-133)
41	Orf4	STNLCTHSFRKKQVR	140-154	no
42	Envelope	TLIVNSVLLFLAFVV	11-25	SARS-CoV-2 (11-25)
43	Envelope	AYCCNIVNVSLVKPT	41-55	SARS-CoV-2 (4-17)
44	Membrane	VPLRGTIVTRPLMES	121-135	SARS-CoV-2 (122-136)
45	Membrane	GHLRMAGHSLGRCDI	146-160	SARS-CoV-2 (147-161)
46	Membrane	KDLPKEITVATSRTL	161-175	SARS-CoV-2 (162-176)
47	Membrane	EITVATSRTLSYYKL	166-180	SARS-CoV-2 (167-181)
48	Membrane	SYYKLGASQRVGTDS	176-189	SARS-CoV-2 (177-187)
49	Membrane	GASQRVGTDSGFAAY	181-195	SARS-CoV-2 (182-196)
50	Membrane	GFAAYNRYRIGNYKL	191-205	SARS-CoV-2 (192-206)
51	Membrane	NRYRIGNYKLNTDHA	196-210	SARS-CoV-2 (197-210)
52	Orf 7	IAEILIIIMRTFRIA	11-25	SARS-CoV-2 (11-22)
53	Orf 7	IWNLDVIISSIVRQL	26-40	SARS-CoV-2 (26-40)
54	Orf 13	ILRLGSQLSLSMARR	46-60	no
55	Orf 13	NLDSLEARAFQSTPI	61-75	no
56	Orf 13	KLATTEELPDEFVVV	81-94	no
57	Nucleocapsid	SPRWYFYYLGTGPEA	106-120	SARS-CoV-2 (105-119)
58	Nucleocapsid	TGPEASLPYGANKEG	116-130	SARS-CoV-2 (115-129)
59	Nucleocapsid	SLPYGANKEGIVWVA	121-135	SARS-CoV-2 (121-134)
60	Nucleocapsid	GETALALLLLDRLNQ	211-225	SARS-CoV-2 (217-229)
61	Nucleocapsid	LLLDRLNQLESKV	223-235	SARS-CoV-2 (222-234)
62	Nucleocapsid	GMSRIGMEV	317-325	SARS-CoV-2 (316-324)
63	Nucleocapsid	WLTYHGAIKLDDKDPQF	331-347	SARS-CoV-2 (330-344)
64	Nucleocapsid	QFKDNVILLNKHIDAYK	346-362	SARS-CoV-2 (350-361)
65	Nucleocapsid	KHIDAYKTFPPTEPK	356-370	SARS-CoV-2 (361-375)
66	Nucleocapsid	YKTFPPTEPKKDKKK	361-375	SARS-CoV-2 (367-381)

no, no aligned sequence in SARS-CoV-2.

### Comparison Between Differential Expression of mRNAs and circRNAs

**Figure 3:**
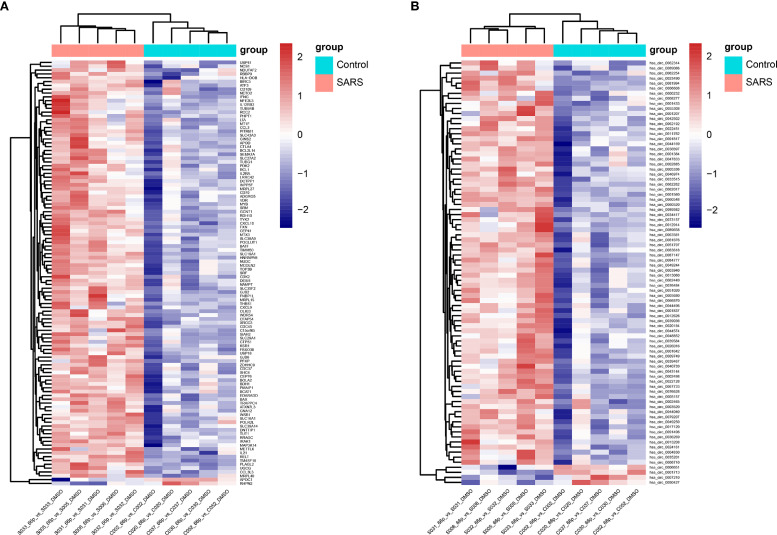
Heatmap of transcriptomic profiling. **(A)** Hierarchical clustering of differential expression of mRNAs. **(B)** Hierarchical clustering of differentially expressed circRNAs.

We randomly thawed PBMCs isolated from convalescent patients with SARS (S005, S006, S031, S032, and S033) and the control group (C002, C020, C030, C037, and C052) from 2018. After stimulating the PBMCs with the 66 peptide pool, Affymetrix^®^ GeneChip was used to identify differentially expressed genes in PBMCs. A total of 111 and 84 differentially expressed mRNAs and circRNAs were identified in the SARS and control groups, respectively. Of the 111 genes, 109 genes were upregulated, whereas 2 genes were downregulated for mRNAs, and of the 84 genes, 80 genes were upregulated and 4 genes were downregulated for circRNAs. The data pertaining to differentially expressed mRNAs and circRNAs between the groups were subjected to unsupervised hierarchical clustering and analysis. The heatmap showed distinguishable mRNA and circRNA expression profiles between the groups (**Figure 3**). The RNAs of the SARS and control groups were clustered together. The top five mRNAs were IFNG, GJB2, THBS1, APOD, and OLIG3, whereas the top five circRNAs were hsa_circ_0034417, hsa_circ_0001642, hsa_circ_0084777, hsa_circ_0009749, and hsa_circ_0067733.

### GO term and KEGG Pathway Enrichment Analyses

**Figure 4:**
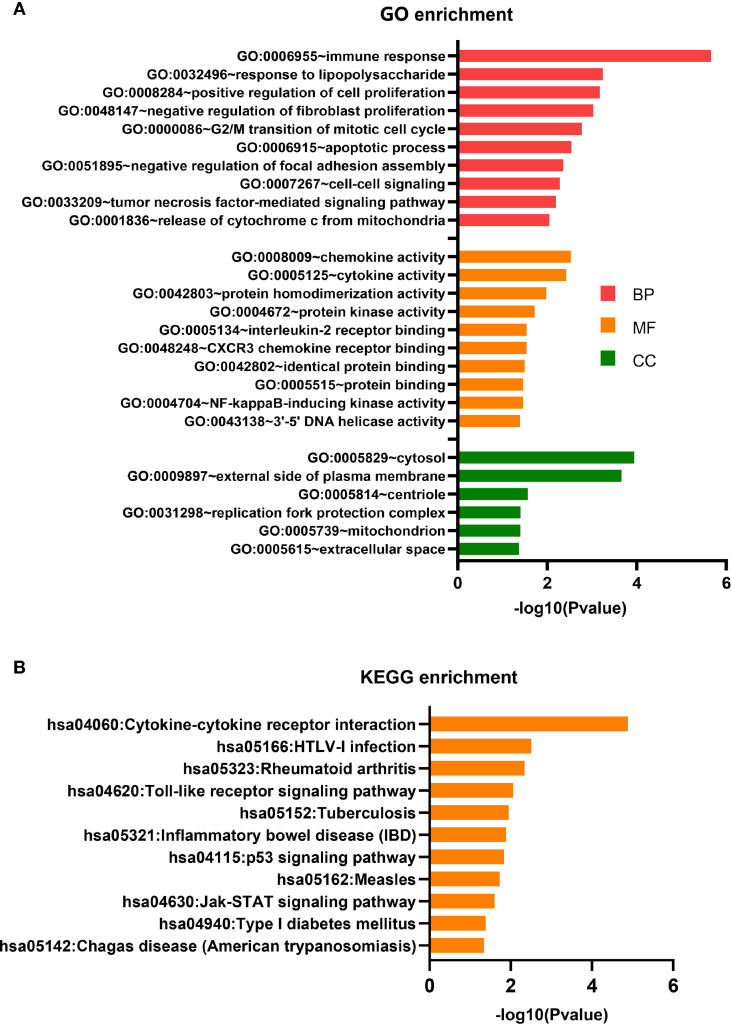
GO terms and KEGG pathway analyses. **(A)** GO term enrichment analysis. **(B)** KEGG pathway enrichment analysis.

The 111 differentially expressed mRNAs (DEMs) were used to investigate GO terms and KEGG pathways using DAVID. In the GO analysis, all enriched terms for the DEMs were ranked by −log10 (P value) as shown. The most significantly enriched GO term in the biological process and molecular functions (**Figure 4A**) was immunity (GO:0006955, GO:0032496, GO:0008009, GO:0005125, GO:0005134, GO:0048248), followed by signal transduction (GO:0007267, GO:0033209, GO:0004672, GO:0004704) and cell growth (GO:0008284, GO:0048147, GO:0000086, GO:0006915). The most significantly enriched GO terms pertaining to cellular components were the cytosol (GO:0005829) and the outer plasma membrane (GO:0009897), which were consistent with the functions of immunity and signal transduction (**Figure 4A**).

The KEGG pathway analysis indicated that 11 KEGG pathways (P < 0.05) were associated with DEMs. The highly enriched pathways targeted by the overexpressed mRNAs were associated with infection and immunity (hsa04060, hsa05166, hsa05323, hsa05152, hsa05321, hsa05162, hsa04940, and hsa05142) (**Figure 4B**). hsa04060 is a cytokine–cytokine receptor interaction pathway that included 11 genes: *IFNG*, *CD70*, *CCL3*, *CXCL9*, *CCL3L3*, *IL2RA*, *LTA*, *CXCL10*, *IL12RB2*, *IL21*, and *RELT.*

### Validation of the Microarray Data Using qPCR

To validate the accuracy and reliability of microarray profiling data, we added PBMC mRNA detection data from convalescent patients with SARS (S016-66, S020-66, S025-66, S044-66, and S046-66) and the corresponding control group (C013, C024, C025, C036, and C047) to verify the Affymetrix GeneChip assay results using qPCR (**Supplementary Table 4** and **Supplementary Figure 1**). The test results of the two methods were relatively consistent.

### Establishment of a Gene Co-Expression Network

A circRNA-miRNA-mRNA regulatory network was constructed to explore the relationship between circRNAs and mRNAs. Finally, 56 mRNAs and 38 circRNAs were found to interact with seven miRNAs (**Supplementary Figure 2**). CircRNAs, which function as miRNA sponges, could competitively bind to miRNAs to influence binding between miRNAs and mRNAs. The cytokine–cytokine receptor interaction pathway (hsa04060) had five genes (*CXCL9*, *IL2RA*, *LTA*, *IL12RB2*, and *RELT*) involved in the network.

## Discussion

### Resource Identification Initiative

We comprehensively analysed humoral, cellular, and molecular level immune responses of convalescent patients with SARS *via* serology, cell experiments, and transcriptome studies on samples collected form patients 15 years post-infection with SARS-CoV. This study yielded certain important findings.

Antibodies displayed immune cross-reactivity between SARS-CoV and SARS-CoV-2. This result was consistent with the results of previous studies. One antibody (S309) from memory B cells of an individual who was infected with SARS-CoV in 2003 potently neutralised SARS-CoV-2 and SARS-CoV pseudoviruses by binding to the spike (S) glycoprotein (Pinto et al., 2020). Twenty serum samples collected from convalescent patients with SARS-CoV during the 2003 SARS outbreak cross-reacted with the S ectodomain, S1, RBD, and S2 proteins of SARS-CoV-2 (Zhu et al., 2020).

Our findings indicated that antibodies targeting the SARS spike protein had persisted 15 years. These results were not entirely consistent with those of previous studies, which had shown that SARS-CoV antibody responses displayed poor longevity. Wu et al. used ELISA based on an inactivated preparation of whole-virus lysate to test SARS convalescent IgG and IgM at different time points. The percentage of IgG positivity was 100% at 91–210 d after the onset of symptoms. However, this value started to decrease, because of which approximately 53% of convalescent patients were positive for IgG at 764–1265 d post-SARS-CoV infection, whereas IgM was negative after 121 d (Wu et al., 2007). Six years post-infection, IgG antibodies specific to SARS-CoV were undetectable in 21 of the 23 convalescent patients, with only two patients maintaining low levels of IgG antibodies, with no detectable peripheral IgG memory B cell responses remaining (Tang et al., 2011). In another study, four convalescent serum samples obtained at month 60 and tested using spike protein-based ELISA were all positive. Neutralising antibody activity was detectable in two convalescent patients (Liu et al., 2011). However, other studies reported that IgG antibodies against SARS-CoV persisted for at least 12 years, where 69.23% (18/26) were positive for IgG in 2015, suggesting the longevity of antibody response (Guo et al., 2020; Sariol and Perlman, 2020).

Only one of the 53 convalescent patients with SARS exhibited serous ACE2 competition positivity. There were indeed no protective antibodies in other individuals, or there were limited antibodies to detect due to insufficient experimental sensitivity.

Serum samples collected from the control population (45 close contacts not infected with SARS-CoV in 2003) did not react with the SARS spike protein. This result was not consistent with that of the study of Ng et al., who reported that of the 50 SARS-CoV-2–uninfected pregnant women sampled in May 2018, five exhibited SARS-CoV-2 S–reactive IgG antibodies, but not IgM or IgA antibodies (Ng et al., 2020). Recent studies indicated that lymphocytes from 20%–50% of unexposed donors displayed significant reactivity with the SARS-CoV-2 antigen (Grifoni et al., 2020; Sette and Crotty, 2020).

GO and KEGG pathway analyses indicated that DEMs were highly enriched in infection, immunity, and signal transduction terms. The DEMs involved included *IFNG*, *CD70*, *CCL3*, *CXCL9*, *CCL3L3*, *IL2RA*, *CXCL10*, *IL21*, and *IL12RB2.* These genes are characterised by high-level production of pro-inflammatory chemokines and cytokines. *IFNG* encodes IFN-γ, a type II interferon. IFN-γ is secreted by cells of both innate and adaptive immune systems. It binds to the IFN-γ receptor to trigger a cellular response to antiviral infections. IFN-γ, which exhibits important immunoregulatory functions, is a potent activator of macrophages. It potentiates antiviral and anti-tumour effects of type I interferons (Shang and Tomasi, 2006; Grant et al., 2021). *CD70* encodes a cytokine that belongs to the tumour necrosis factor ligand family. This cytokine acts as a ligand for CD27, where the CD70/CD27 pathway plays an important role in the generation and maintenance of T cell immunity, with particular reference to antiviral responses. CD27 induces the proliferation of co-stimulated T cells, enhances the generation of cytolytic T cells, and promotes T cell activation (Izawa et al., 2017). *CCL3* encodes macrophage inflammatory protein 1 alpha that plays a role in the inflammatory response by binding to the receptors CCR1, CCR4, and CCR5 (Schwarz et al., 1997). *CCL3L3* encodes cytokines, which are ligands of CCR1, CCR3, and CCR5 that are chemotactic for lymphocytes and monocytes (Gaudet et al., 2011). The cytokine encoded by *CXCL9* affect the movement and activation of T cells by binding to CXCR3 (Lasagni et al., 2003). *CXCL10* encodes the chemokine IFN-γ-inducible protein-10 (IP-10). Binding of IP-10 to the CXCR3 receptor activates G protein-mediated signalling, which results in downstream activation of the phospholipase C-dependent pathway, resulting in increased intracellular calcium production and actin reorganisation, which in turn, stimulate the migration of monocytes, natural killer cells, and T cells, and modulation of adhesion molecule expression. Thus, IP-10 plays an important role during viral infections (Lasagni et al., 2003). The short peptides we synthesised overlapped with the proteins of SARS-CoV-2 and our findings pertaining to these chemokines and cytokines also overlapped with those described in previous studies on SARS and the current COVID-19 pandemic. The IFN-γ level in the sera of patients with SARS was higher than that in the control group (Zhang et al., 2004). *IFNG* expression was detected in the T cells from bronchoalveolar lavage fluid of patients with COVID-19 (Grant et al., 2021). Analysis of differential levels of proteins in COVID-19 cases compared with those in healthy controls revealed 21- and 9-fold increase in IFN-γ and CXCL, respectively (Sims et al., 2021). IP-10 has been implicated in the pathogenesis of ARDS in mouse SAR-CoV infection models (Glass et al., 2004). Increased plasma concentrations of IP-10 occurred in 88% of patients (Tang et al., 2005). Monocyte-derived macrophages (moMa) showed high expression of the chemokine encoding gene, *CXCL10* (Chua et al., 2020). The plasma IP-10 levels are strongly associated with disease severity as well as COVID-19 prognosis (Yang et al., 2020b). A significant upsurge in the chemokine CCL3 was observed in the SARS-CoV-infected group at 24 h, and this upsurge was in line with the elevated infiltration of T cells, NK cells, and monocytes (Yao et al., 2020). Both non-resident macrophages and moMa in critical patients with COVID-19 showed a high expression of the chemokine encoding gene, *CCL3* (Chua et al., 2020) The excessive release of the chemokine CXCL9 in patients with COVID-19 with severe clinical presentations was significantly higher compared with those of patients with mild and moderate clinical presentations (Jain et al., 2021).

The current study was beset with three main limitations. First, we could not use live SARS-CoV-2 to detect cross-reactive neutralisation of the sera of convalescent patients with SARS. Second, due to the small number of isolated PBMCs, samples from different convalescent patients were used in different experiments, and flow cytometric immune cell classification test was not performed. The clinical characteristics of the population of samples used in the experiments of ELISPOT and GeneChip were compared in **Supplementary Tables 5**, **6**. Third, neither our competing endogenous RNA network analysis nor our literature review identified any meaningful circRNAs and miRNAs associated with COVID-19, and no further research was conducted along these lines.

In conclusion, SARS-CoV and SARS-CoV-2 share genomes and proteomes. Our study revealed that antibodies from convalescent patients with SARS persisted for 15 years and displayed immune cross-reactivity with the spike protein of SARS-CoV-2. DEMs detected *via* Affymetrix GeneChip showed that SARS elicits cytokine and chemokine responses, partially similar to previously published data about COVID-19. Thus, the immune status of convalescent patients with SARS 15 years post-infection may provide a reference for managing COVID-19.

## Data Availability Statement

The raw data supporting the conclusions of this article will be made available by the authors, without undue reservation.

## Ethics Statement

The studies involving human participants were reviewed and approved by Ethics Committee of Peking University People’s Hospital (2018PHB010-01). The patients/participants provided their written informed consent to participate in this study.

## Author Contributions

ZG and BJ designed the study. LZ completed the cytology experiment and drafted the manuscript. NH and BJ organised the examination of convalescent patients with SARS. YZ designed the 66 epitope peptides pool. YZ and YX verified the underlying data. HR and HC completed the antibody testing. JL, YC, and BY collected the clinical data. ZG, BJ, and YZ obtained research funding. All authors contributed to the article and approved the submitted version.

## Funding

This work was supported by grants from the Chinese Science and Technology Key Project [2017ZX10103004-006], the National Key Research and Development Programme of China [2016YFC0903800], the Emergency Scientific Research Project of Novel Coronavirus Prevention and Control from Xiamen University [20720200017, 20720200032]. The funders had no role in the study design, data collection and analysis, decision to publish, or preparation of the manuscript.

## Conflict of Interest

The authors declare that the research was conducted in the absence of any commercial or financial relationships that could be construed as a potential conflict of interest.

## Publisher’s Note

All claims expressed in this article are solely those of the authors and do not necessarily represent those of their affiliated organizations, or those of the publisher, the editors and the reviewers. Any product that may be evaluated in this article, or claim that may be made by its manufacturer, is not guaranteed or endorsed by the publisher.
