# Supplementary material for: Detection of Antibodies Against the SARS-CoV-2 Spike Protein and Analysis of the Peripheral Blood Mononuclear Cell Transcriptomic Profile, 15 Years After Recovery From SARS
Source: Front Cell Infect Microbiol. 2021 Nov 18;11:768993. doi: 10.3389/fcimb.2021.768993 (PMC8636717; doi:10.3389/fcimb.2021.768993)
Supplement: Supplementary file 1 [file DataSheet_1.docx]

Supplementary Material

**Supplementary Table1.** **Immune cross-reactivity of convalescent serums against the SARS-CoV-2 spike protein collected from patients with SARS 15 years later**

| SARS | | | Control | | |
| --- | --- | --- | --- | --- | --- |
| Number | **IgM (S/CO)** | **IgG (S/CO)** | **Number** | **IgM (****S/CO)** | **IgG (S/CO)** |
| S01 | 0.11 | 2.45 | **C01** | 0.16 | 0.07 |
| S02 | 0.10 | 0.00 | **C02** | 0.09 | 0.18 |
| S03 | 0.09 | 0.93 | **C03** | 0.15 | 0.00 |
| S04 | 0.11 | 0.00 | **C04** | 0.10 | 0.04 |
| S05 | 0.12 | 6.80 | **C05** | 0.10 | 0.13 |
| S06 | 0.15 | 0.00 | **C13** | 0.13 | 0.13 |
| S08 | 0.11 | 0.53 | **C14** | 0.90 | 0.08 |
| S09 | 0.09 | 1.76 | **C15** | 0.10 | 0.06 |
| S10 | 0.10 | 10.81 | **C16** | 011 | 0.05 |
| S12 | 0.11 | 6.08 | **C19** | 0.09 | 0.06 |
| S13 | 0.11 | 2.14 | **C20** | 0.13 | 0.04 |
| S14 | 0.21 | 3.26 | **C21** | 0.09 | 0.04 |
| S15 | 0.12 | 5.14 | **C22** | 0.11 | 0.11 |
| S16 | 0.13 | 5.27 | **C23** | 0.15 | 0.08 |
| S17 | 0.24 | 0.00 | **C24** | 0.10 | 0.18 |
| S18 | 0.49 | 0.00 | **C25** | 0.12 | 0.06 |
| S19 | 0.11 | 1.05 | **C26** | 0.10 | 0.07 |
| S20 | 0.12 | 5.67 | **C27** | 0.16 | 0.05 |
| S21 | 0.10 | 0.00 | **C28** | 0.11 | 0.09 |
| S22 | 0.10 | 17.24 | **C29** | 0.09 | 0.00 |
| S23 | 0.26 | 1.35 | **C30** | 0.12 | 0.06 |
| S24 | 0.12 | 3.31 | **C33** | 0.13 | 0.00 |
| S25 | 0.09 | 0.06 | **C34** | 0.23 | 0.06 |
| S26 | 0.17 | 4.84 | **C35** | 0.10 | 0.09 |
| S27 | 0.10 | 7.00 | **C36** | 0.14 | 0.06 |
| S28 | 0.12 | 0.00 | **C37** | 0.09 | 0.12 |
| S29 | 0.13 | 0.63 | **C38** | 0.10 | 0.04 |
| S30 | 0.11 | 0.71 | **C39** | 0.12 | 0.05 |
| S31 | 0.13 | 0.74 | **C40** | 0.10 | 0.00 |
| S32 | 0.15 | 3.18 | **C42** | 0.12 | 0.00 |
| S33 | 0.29 | 0.00 | **C43** | 0.09 | 0.00 |
| S34 | 0.15 | 7.69 | **C44** | 0.10 | 0.00 |
| S35 | 0.21 | 0.00 | **C45** | 0.12 | 0.09 |
| S36 | 0.10 | 0.00 | **C46** | 0.10 | 0.03 |
| S38 | 0.13 | 0.00 | **C47** | 0.10 | 0.00 |
| S39 | 0.12 | 0.00 | **C48** | 0.10 | 0.00 |
| S41 | 0.47 | 0.00 | **C49** | 0.08 | 0.05 |
| S42 | 0.14 | 1.11 | **C50** | 0.10 | 0.77 |
| S43 | 0.11 | 1.61 | **C51** | 0.19 | 0.05 |
| S44 | 0.10 | 5.56 | **C52** | 0.17 | 0.00 |
| S45 | 0.16 | 0.00 | **C53** | 0.09 | 0.07 |
| S46 | 0.14 | 0.00 | **C54** | 0.09 | 0.05 |
| S47 | 0.14 | 0.39 | **C55** | 0.09 | 0.00 |
| S48 | 0.12 | 3.24 | **C56** | 0.10 | 0.04 |
| S49 | 0.13 | 2.7 | **C57** | 0.08 | 0.02 |
| S50 | 0.14 | 10.45 |  |  |  |
| S51 | 0.46 | 2.79 |  |  |  |
| S52 | 0.14 | 3.17 |  |  |  |
| S53 | 0.14 | 0.31 |  |  |  |
| S54 | 0.11 | 2.23 |  |  |  |
| S56 | 0.12 | 1.12 |  |  |  |
| S57 | 0.12 | 0.00 |  |  |  |
| S60 | 0.15 | 2.00 |  |  |  |
| SARS group: convalescent patients who were infected with SARS-CoV in 2003; Control group: close contacts of the SARS group patients who were not infected with SARS-CoV in 2003. Serum samples were collected in 2018, 15 years after infection; S01–S60: different individuals in the SARS group; C01–C57: different individuals in the Control group; S/CO: signal-to-cutoﬀ ratio | | | | | |

**Supplementary Table 2. SARS-CoV-2 neutralizing antibody test by competitive inhibition ELISA**

| SARS | | | | Control | | |
| --- | --- | --- | --- | --- | --- | --- |
| Number | **OD450** | **Judgement**  **Cut off = 0.274** | **Number** | | **OD450** | **Judgement**  **Cut off = 0.274** |
| S01 | 0.404 | N | | **C01** | 0.346 | N |
| S02 | 0.476 | N | | **C02** | 0.387 | N |
| S03 | 0.431 | N | | **C03** | 0.358 | N |
| S04 | 0.49 | N | | **C04** | 0.379 | N |
| S05 | 0.488 | N | | **C05** | 0.466 | N |
| S06 | 0.561 | N | | **C13** | 0.414 | N |
| S08 | 0.565 | N | | **C14** | 0.334 | N |
| S09 | 0.593 | N | | **C15** | 0.364 | N |
| S10 | 0.301 | N | | **C16** | 0.399 | N |
| S12 | 0.307 | N | | **C19** | 0.342 | N |
| S13 | 0.373 | N | | **C20** | 0.306 | N |
| S14 | 0.447 | N | | **C21** | 0.340 | N |
| S15 | 0.398 | N | | **C22** | 0.318 | N |
| S16 | 0.403 | N | | **C23** | 0.360 | N |
| S17 | 0.458 | N | | **C24** | 0.401 | N |
| S18 | 0.431 | N | | **C25** | 0.367 | N |
| S19 | 0.387 | N | | **C26** | 0.387 | N |
| S20 | 0.334 | N | | **C27** | 0.539 | N |
| S21 | 0.388 | N | | **C28** | 0.524 | N |
| S22 | **0.171** | **P** | | **C29** | 0.465 | N |
| S23 | 0.405 | N | | **C30** | 0.501 | N |
| S24 | 0.480 | N | | **C33** | 0.439 | N |
| S25 | 0.439 | N | | **C34** | 0.405 | N |
| S26 | 0.411 | N | | **C35** | 0.521 | N |
| S27 | 0.310 | N | | **C36** | 0.395 | N |
| S28 | 0.336 | N | | **C37** | 0.389 | N |
| S29 | 0.351 | N | | **C38** | 0.456 | N |
| S30 | 0.439 | N | | **C39** | 0.327 | N |
| S31 | 0.417 | N | | **C40** | 0.362 | N |
| S32 | 0.360 | N | | **C42** | 0.446 | N |
| S33 | 0.384 | N | | **C43** | 0.378 | N |
| S34 | 0.335 | N | | **C44** | 0.558 | N |
| S35 | 0.335 | N | | **C45** | 0.426 | N |
| S36 | 0.365 | N | | **C46** | 0.492 | N |
| S38 | 0.387 | N | | **C47** | 0.412 | N |
| S39 | 0.390 | N | | **C48** | 0.394 | N |
| S41 | 0.375 | N | | **C49** | 0.371 | N |
| S42 | 0.376 | N | | **C50** | 0.437 | N |
| S43 | 0.399 | N | | **C51** | 0.532 | N |
| S44 | 0.385 | N | | **C52** | 0.511 | N |
| S45 | 0.395 | N | | **C53** | 0.410 | N |
| S46 | 0.394 | N | | **C54** | 0.439 | N |
| S47 | 0.379 | N | | **C55** | 0.394 | N |
| S48 | 0.444 | N | | **C56** | 0.388 | N |
| S49 | 0.378 | N | | **C57** | 0.325 | N |
| S50 | 0.399 | N | |  |  |  |
| S51 | 0.377 | N | |  |  |  |
| S52 | 0.516 | N | |  |  |  |
| S53 | 0.404 | N | |  |  |  |
| S54 | 0.440 | N | |  |  |  |
| S56 | 0.408 | N | |  |  |  |
| S57 | 0.403 | N | |  |  |  |
| S60 | 0.309 | N | |  |  |  |
| SARS group: convalescent patients who were infected with SARS-CoV in 2003; Control group: close contacts of the SARS group patients who were not infected with SARS-CoV in 2003. S01–S60: different individuals in the SARS group; C01–C57: different individuals in the Control group. Cut-off = 1/2ODS0 = 0.274. S0 is the average absorbance value of the 0 ng/ml standard. OD of sample < 1/2ODS0 was considered positive (P); OD sample > 1/2ODS0 was considered negative (N). | | | | | | |

**Supplementary Table 3. Logistic univariate and multivariate analyses of the characteristics for IgG positivity using a microsphere-based antibody assay**

|  | Univariate analysis |  | Multivariate analysis |  |
| --- | --- | --- | --- | --- |
|  | **Odds ratio (95% CI)** | **P value** | **Odds ratio (95% CI)** | **P value** |
| Male sex | 0.438 (0.093-2.063) | 0.297 |  |  |
| Age | 1.004 (0.943-1.068) | 0.912 |  |  |
| Duration of fever(days) | 1.007 (0.941-1.078) | 0.837 |  |  |
| Rigor | 0.285 (0.086-0.958) | **0.042** |  |  |
| Myalgia | 0.338 (0.101-1.134) | 0.079 |  |  |
| Cough | 0.929(0.309-2.790) | 0.895 |  |  |
| Dyspnea | 0.824 (0.267-2.540) | 0.735 |  |  |
| Vomiting | 0.318 (0.058-1.756) | 0.189 |  |  |
| Diarrhea | 0.880 (0.207-3.742) | 0.863 |  |  |
| Methylprednisolone | 1.8375(0.438-4.318) | 0.585 |  |  |
| Immunoglobulin | 0.515 (0.161-1.646) | 0.263 |  |  |
| Thymosin | 0.286 (0.085-0.958) | **0.042** |  |  |
| ARDS | 0.400 (0.105-1.526) | 0.180 |  |  |
| Lung injury | 0.674(0.212–2.142) | 0. 503 |  |  |
| Bone injury | 2.4 (0.665–8.666) | 0.181 |  |  |
| SCAP | 1.012 (0.338–3.033) | 0.983 |  |  |

**Supplementary Table 4. qPCR primers for verification**

| **Gene name** | **Forward primer** | **Reverse primer** |
| --- | --- | --- |
| **IFNG** | TGAATGTCCAACGCAAAGCA | CTGGGATGCTCTTCGACCTC |
| **CD70** | GGGGCAGCTACGTATCCATC | TGGGAGGCAATGGTACAACC |
| **IL12RB2** | ACGGAGTTCTATACCAGAGTTGA | AGTCACATCGCCTCTCTTGC |
| **CCL3** | ATTCCGTCACCTGCTCAGAA | TGGCTGCTCGTCTCAAAGTA |
| **CXCL9** | ATTGGAGTGCAAGGAACCCC | ATTTTCTCGCAGGAAGGGCT |
| **CXCL10** | AGCAGAGGAACCTCCAGTCT | ATGCAGGTACAGCGTACAGT |
| **IL2RA** | AGGGCTCTACACAGAGGTCC | GTGACGAGGCAGGAAGTCTC |
| **CCL3L3** | ATGCAGGTCTCCACTGCTGC | CGTCTCAAAGTAGTCAGCTATG |
| **LTA** | TTCGTGCTTTGGACTACCGC | CACACACCCTTGGGAGGAAG |
| **IL21** | CACCTTCCACAAATGCAGGG | TCTGGATAGGTAAAGATAAAGCAGA |
| **RELT** | ATTAGCCACACCCTTGCCTC | GGACTCACTCTCCTAGCCCA |
| **GAPDH** | CTCCAAAATCAAGTGGGGCG | TGGTTCACACCCATGACGAA |

**Supplementary Table 5. Clinical** **characteristics of population of patients whose samples were used in the ELISPOT assay and GeneChip array**

|  | ELISPOT  n=5 | GeneChip  n=5 | P value |
| --- | --- | --- | --- |
| Male sex (%) | 1 (20%) | 0 (0%) | 1.000 |
| Age (years) | 44.6 ± 2.0 | 44.4 ± 3.3 | 0.960 |
| Clinical symptoms, n (%) |  |  |  |
| Fever | 5 (100%) | 5 (100%) | - |
| Duration of fever(days) | 11.75 ± 1.97 | 17.75 ± 7.61 | 0.474 |
| Rigor | 1 (80%) | 3 (40%) | 0.524 |
| Myalgia | 0 (0.0%) | 2 (40%) | 0.444 |
| Cough | 3 (60%) | 3 (60%) | 1.000 |
| Dyspnea | 2 (40%) | 3 (60%) | 1.000 |
| Vomiting | 0 (0%) | 1 (20%) | 1.000 |
| Diarrhea | 0 (0%) | 1 (20%) | 1.000 |
| Treatments |  |  |  |
| Total Methylprednisolone (mg) | 2000 ± 898 | 1328 ± 662 | 0.559 |
| Immunoglobulin | 1 (20%) | 3 (60%) | 0.524 |
| Thymosin | 2 (40%) | 3 (60%) | 1.000 |
| Severity |  |  |  |
| ARDS | 0 (0%) | 0 (0%) | - |
| Lung injury | 1 (20%) | 2(40%) | 1.000 |
| Bone injury | 3 (60%) | 2 (40%) | 1.000 |
| SCAP | 1 (20%) | 1 (20%) | 1.000 |
| Data are presented as mean ± standard error from the mean, or median (interquartile range) or n (%). ARDS, acute respiratory distress syndrome; SCAP, severe community acquired pneumonia | | | |

**Supplementary Table 6. Clinical characteristics compare of population for ELISPOT assay and GeneChip array**

|  | ELISPOT (SARS)  n=5 | GeneChip (SARS)  n=5 | ELISPOT (Control)  n=5 | GeneChip (Control)  n=5 | P value |
| --- | --- | --- | --- | --- | --- |
| Male sex (%) | 1 (20%) | 0 (0.0%) | 3 (60%) | 2(40%) | 0.19 |
| Age (years) | 44.6 ± 2.0 | 44.4 ± 3.3 | 43.6 ± 1.9 | 45.6 ± 1.2 | 0.936 |
| Laboratory findings |  |  |  |  |  |
| White blood cell (×10^3^/mm^3^) | 5.56 ± 0.64 | 4.94 ± 0.87 | 5.86 ± 0.71 | 6.01 ± 0.63 | 0.733 |
| Neutrophils(×10^3^/mm^3^) | 3.01 ±0.31 | 2.61 ± 0.46 | 2.85 ± 0.39 | 3.15 ± 0.42 | 0.802 |
| Lymphocytes(×10^3^/mm^3^) | 2.00 ± 0.41 | 1.96 ± 0.43 | 2.42 ± 0.34 | 2.31 ± 0.26 | 0.762 |
| Hemoglobin level (g/dL) | 134.80 ± 7.96 | 137.00 ± 3.52 | 149.00 ±7.06 | 147.00 ± 6.92 | 0.356 |
| Platelet count (×10^3^/mm^3^) | 289.60 ± 27.74 | 271.60 ± 36.37 | 234.80 ± 17.43 | 253.20 ± 39.03 | 0.644 |
| Glucose (mmol/L) | 5.10 ± 0.21 | 5.23 ± 0.21 | 5.41 ± 0.29 | 5.51 ± 0.31 | 0.682 |
| Albumin (g/L) | 45.72 ± 0.96 | 45.48 ± 0.36 | 47.04 ± 1.55 | 45.46 ± 1.91 | 0.811 |
| ALT (U/L) | 17.00 ± 3.67 | 13.40 ± 1.81 | 23.60 ± 4.18 | 23.00 ± 2.76 | 0.116 |
| AST (U/L) | 16,80 ± 1.85 | 17.60 ± 1.33 | 22.60 ± 2.42 | 21.20 ± 2.69 | 0.206 |
| LDH (U/L) | 150.00 ± 6.73 | 156.80 ± 17.99 | 164.00 ± 10.04 | 161.60 ± 10.13 | 0.849 |
| CREA (µmol/L) | 60.80 ± 7.39 | 56.80 ± 4.28 | 65.00 ± 1.92 | 62.20 ± 3.89 | 0.682 |
| IgA (g/L) | 2.58 ± 0.62 | 2.62 ± 0.51 | 2.31 ± 0.50 | 1.87 ± 0.40 | 0.719 |
| IgG (g/L) | 10.90 ± 0.43 | 12.08 ± 1.26 | 14.64 ± 1.21 | 13.28 ± 1.28 | 0.138 |
| IgM (g/L) | 1.14 ± 0.19 | 1.15 ± 0.21 | 1.03 ± 0.13 | 1.00 ± 0.15 | 0.898 |
| C3 (g/L) | 0.94 ± 0.05 | 0.83 ± 0.06 | 0.93 ± 0.10 | 1.02 ± 0.03 | 0.291 |
| Data are presented as means ± standard error from the mean, or median (interquartile range) or n (%). ARDS, acute respiratory distress syndrome; SCAP, severe community acquired pneumonia | | | | | |
