# Supplementary figures and images for: Detection of Antibodies Against the SARS-CoV-2 Spike Protein and Analysis of the Peripheral Blood Mononuclear Cell Transcriptomic Profile, 15 Years After Recovery From SARS
Source: Front Cell Infect Microbiol. 2021 Nov 18;11:768993. doi: 10.3389/fcimb.2021.768993 (PMC8636717; doi:10.3389/fcimb.2021.768993)

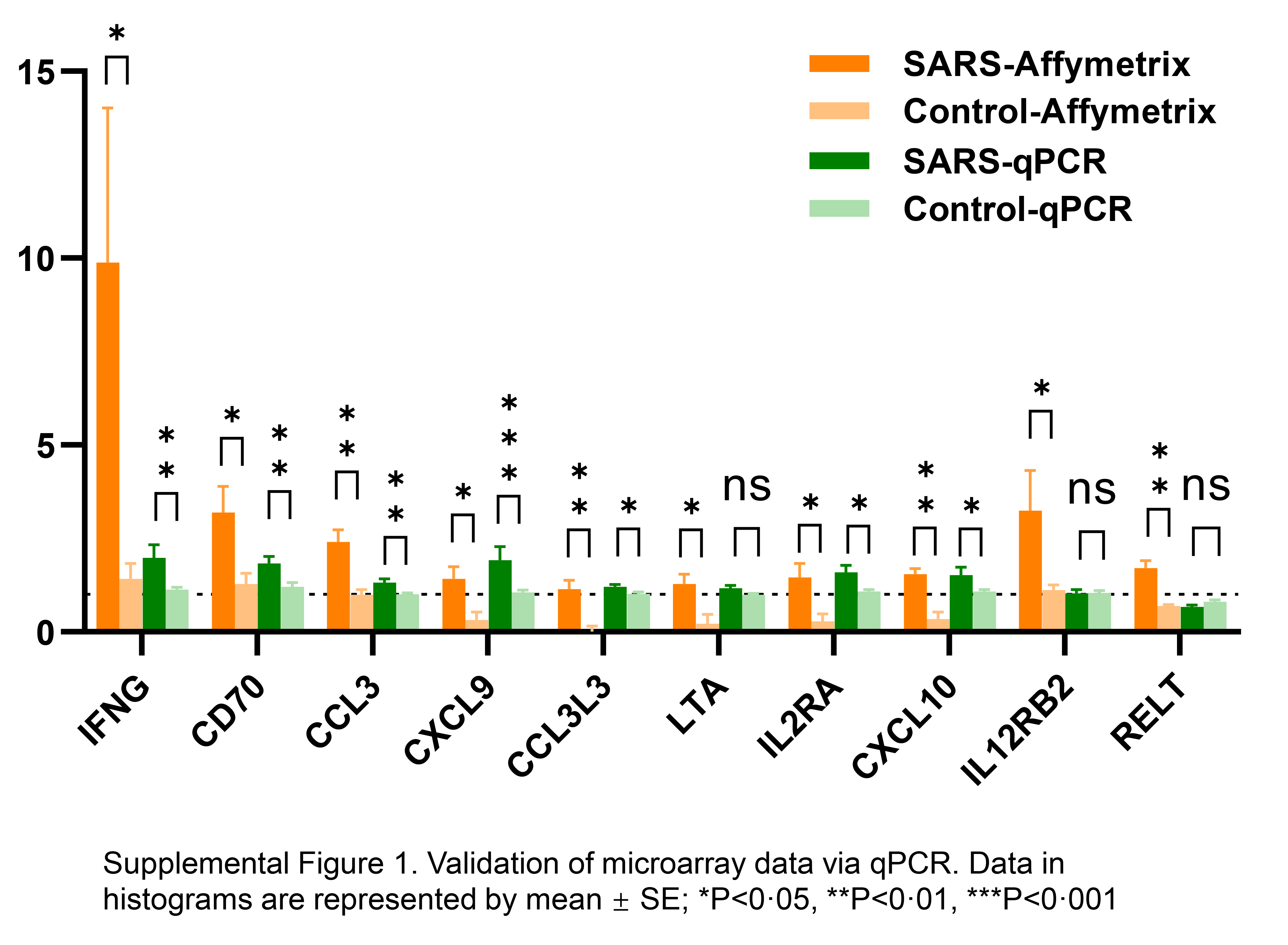

Supplement: Supplementary file 2 [file Image_1.tif]

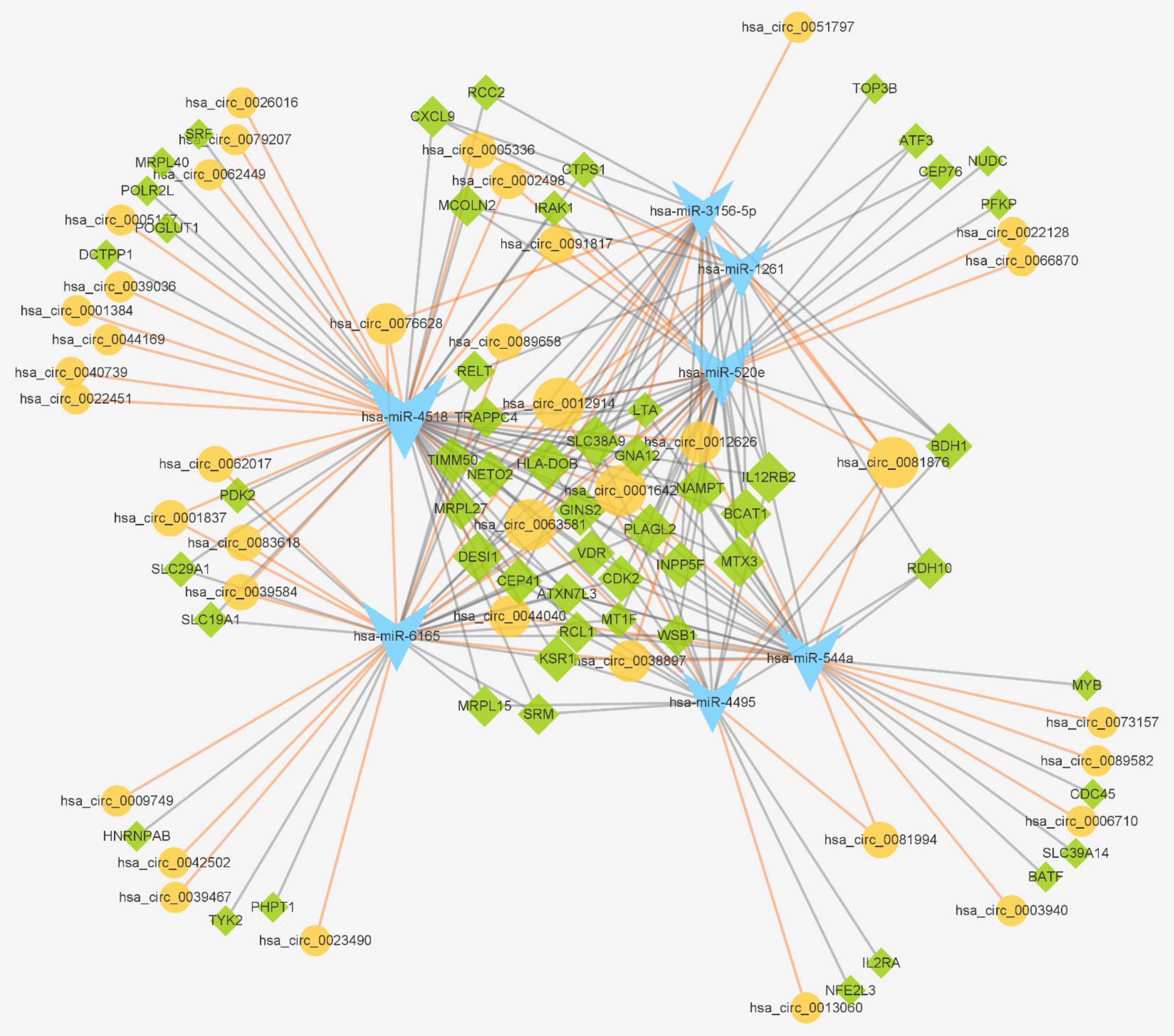

Supplement: Supplementary file 3 [file Image_2.tif]
